# Based on Molecular Subtypes, Immune Characteristics and Genomic Variation to Constructing and Verifying Multi-Gene Prognostic Characteristics of Colorectal Cancer

**DOI:** 10.3389/fcell.2022.828415

**Published:** 2022-02-23

**Authors:** Lei Gu, Chunhui Jiang, Chunjie Xu, Ye Liu, Hong Zhou

**Affiliations:** Department of Gastrointestinal Surgery, Renji Hospital, School of Medicine, Shanghai Jiao Tong University, Shanghai, China

**Keywords:** COAD, molecular subtype, TCGA, multi-gene signature, prognosis

## Abstract

**Background:** Colon cancer (COAD) has been identified as being among the most prevalent tumors globally and ranked the third major contributor to cancer-related mortality. COAD is a molecularly heterogeneous disease. There are great differences in clinical manifestations and prognosis among different molecular subtypes.

**Methods:**379 TCGA-COAD samples were divided into four subtypes: primary proliferative, with collective, crypt-like, and EMT invasion. The differences among the four subtypes were analyzed from the multidimensional perspectives of immunity, genomic variation, and prognosis. The limma package was utilized to identify differentially expressed genes (DEGs) amongst different molecular subtypes. Phenotype-related coexpressed gene modules were identified using WGCNA. The polygenic prognosis model was created utilizing the lasso Cox analysis and verified by time-dependent subject operating characteristics (ROC).

**Results:** There are some differences in prognosis, TMB and common gene variation, immune score, and immunotherapy/chemotherapy between proliferative and three invasive molecular subtypes. 846 differential genes (DEGs) were obtained by limma packet analysis. Differential gene analysis was utilized to screen the DEGs among distinct subtypes, which were significantly enriched in the pathways related to tumorigenesis and development. Co-expression network analysis found 46 co-expressed genes correlated with proliferative and three invasive phenotypes. Based on differentially co-expressed genes, we developed a prognostic risk model of 8-genes signature, which exhibited strong stability regardless of external and internal validation. RT-PCR experiments proved the expression of eight genes in tumor and normal samples.

**Conclusion:** We have developed an eight-gene signature prognostic stratification system. Furthermore, we proposed that this classifier can serve as a molecular diagnostic tool to assess the prognosis of colon cancer patients.

## Introduction

Colon cancer is extensively recognized to be among the most prevalent tumors globally and has been ranked as the third major contributor to cancer mortality (Labianca et al., 2010). COAD is the main pathological type of colon cancer. A growing body of data indicates that COAD is a molecularly diverse illness in which unique molecular alterations influence the growth and survival of tumor cells, as well as their differentiation, apoptosis, and distant metastasis (Comprehensive molecular c, 2012). The conventional TNM staging may not be able to evaluate its metastatic potential because of the unique heterogeneity of COAD, and its staging may not be completely positively correlated with survival (Chu et al., 2016). Although the survival rate of COAD patients has been improved with the increase of treatment methods, the prognosis is still very poor (Neri et al., 2010; Roncucci and Mariani, 2015).In addition to targeted therapy, immunotherapy seems to be a promising treatment for advanced CRC. Recent research has demonstrated that immunotherapy could be beneficial for colon cancer patients who have DMMR/MSI-H (Asaoka et al., 2015; Overman et al., 2017), and the benefit is not obvious in MSS/MSI-L patients. Unfortunately, the proportion of DMMR/MSI-H in metastatic colon cancer is only about 5% (Jung et al., 2020), and the effective rate in such patients is only 30–40% (Le et al., 2017), which indicates that there are some limitations in the application of MSI status as an immune checkpoint inhibitor. On the other hand, it shows that due to the obvious heterogeneity of advanced CRC, a single molecular expression state may not be enough to reflect the information of the whole tumor. Therefore, it is urgent to explore a reliable biomarker or prognostic system, and further clarify the specific information of COAD typing, so as to provide the basis for clinical individualized treatment.

According to the spontaneous model of canine colorectal cancer and the characteristics of human CRC, Shayingzhao et al. divided CRC into the proliferative type and metastatic type, in which the metastatic type was specifically divided into collective type, crypt-like type, and EMT. These four subtypes have their unique biological behavior, pathway activation, and molecular mutation. Proliferative type shows that abnormal Wnt/β-catenin signaling pathway activation results in cell cycle and proliferation, which is the most significant feature of proliferative tumors. In a series of mechanisms, epithelial cells may be changed into cells with a mesenchymal phenotype in a process known as epithelial-mesenchymal transformation (EMT). This transformation makes it easier for tumor cells to separate from the primary tissue and metastasize (Thiery, 2002; Friedl and Gilmour, 2009). Collective invasion is defined as the migration of a group of cells while maintaining intercellular contact. These cells are usually epithelial. CryptLike invasion refers to the CryptLike invasion of cancer cells through CryptLike structures. At present, the research is limited. Usually, these cancer cells are MYC positive, similar to crypt stem cells or progenitor cells. The researchers believe that due to the significant proliferation of fibroblasts, they can develop crypts in non-mucosal sites, making the microenvironment more like mucosa (Wang et al., 2018).In this study, only the pathway and some specific molecular mutation characteristics of each subtype were preliminarily analyzed, but there was no overall comprehensive analysis of the prognostic characteristics and potential therapeutic targets of each subtype.

In this study, according to the molecular typing determined by Zhao et al., we analyzed the differences between the proliferative and three invasive molecular subtypes in prognosis, TMB and common gene variation, immune score, and the efficacy of immunotherapy/chemotherapy.The co-expressed genes related to proliferative phenotype and three invasive phenotypes were identified by co-expression network analysis. Based on the differentially co-expressed genes, we built the prognostic risk model of 8-gene signature (SPARCL1, HAND1, CRIP2, ZNF385A, CXCL1, CLEC10A, PTGS1, and PTN). Regardless of external and internal validation, the 8-gene signature has strong stability, indicating that our risk prediction model can play a stable predictive effect. Based on this, we provide a stable prognostic model for colorectal cancer and provide a basis for individualized treatment of different subtypes of COAD.

## Materials and Methods

### Data Sources

TCGA GDC API was employed to obtain the RNA SEQ data of TCGA-COAD. After screening, we included a sum of 343 samples. In addition, we downloaded the GSE17538 chip data set in conjunction with survival duration from the Gene Expression Omnibus (GEO) database and finally included 226 samples. The subtypes of TCGA-COAD were screened from the Supplementary Materials studied by Shaying Zhao et al. (Wang et al., 2018), after deleting redundant samples through the sample information in the ‘case’ column, a total of 366 samples were divided into four subtypes, of which 155 samples were collective subtypes, 76 samples were crypt-like subtypes, 62 samples were EMT subtypes, and 73 samples were identified as proliferative subtypes. As shown in Supplementary Table S1.

### Data Preprocessing

#### TCGA Data Preprocessing

The RNA-seq data of TCGA were preprocessed in the steps below to obtain 343 colon cancer samples:

1) Extraction of primary colon cancer samples.
2) Patients having a survival duration of more than 30 days and a good survival status were included.
3) Patients with molecular typing results in the study of Shaying Zhao et al.
4) The expression profiles of 25483 genes were obtained by matching ENSG to GeneSymbol.

#### GEO Data Preprocessing

Preprocess geo dataset GSE17538 in several steps:

1) Download the standardized probe expression profile data of GSE17538 chip data from the GEO database;
2) According to the annotation file of the platform corresponding to each chip data, convert the expression profile at the probe level into the expression profile at the gene level. In this regard, when several probes correspond to the very same gene, the gene expression should be calculated using the average of the multiple probes, and the probe should be removed in the case where only one probe corresponds to multiple genes;
3) The samples of primary colon cancer were extracted, and the patients having a survival duration of more than 30 days and a good survival status were included in the present study. Finally, GSE17538 included 229 samples.

### Relationship Between Proliferative and Invasive Molecular Subtypes and TMB and Common Gene Variants

Further, we explored whether there were differences in genomic data between molecular subtypes. We acquired the mutation data set and copy number variation data processed by mutect2 software of TCGA-COAD. Then, the Fisher test was employed to screen the genes that have a substantial mutation, significant copy deletion, and significant copy amplification differences in each subtype.

### Differential Analysis of Proliferative and Invasive Molecular Subtypes in the Efficacy of Immunotherapy/Chemotherapy

We examined the differences among distinct molecular subtypes in immunotherapy and chemotherapy. TIDE(http://tide.dfci.harvard.edu/) Software was utilized to evaluate the potential clinical effects of immunotherapy in proliferative and invasive molecular subtypes. The higher the tide prediction score, the higher the possibility of immune escape, suggesting that the patient is less likely to benefit from immunotherapy.

### Identify Phenotype Related Coexpressed Gene Modules

We used the WGCNA module in R software(Langfelder and Horvath, 2008) to detect phenotype-related co-expression modules. Specifically, we selected TCGA expression profile data set and screened genes having MAD more than 50 percent as gene expression profiles. Firstly, we clustered the samples together and screened them for co-expression modules. In the study, we discovered that the co-expression network corresponds to the scale-free network, indicating that the log(k) of the node with linkage degree k is inversely associated with the log(P (k), which is the probability of the node, and the correlation coefficient is higher than 0.85. We then transformed an adjacency matrix from an expression matrix, and subsequently transformed the adjacency matrix into a topology matrix. Subsequently, for the purpose of clustering genes, we utilized the average linkage hierarchical clustering technique as per the standards of a hybrid dynamic cut tree with the aid of Tom. We also specified 30 as the limit quantity of genes present in each gene network module. After determining the gene modules utilizing the dynamic cutting approach, we computed the eigengenes of each module one at a time and clustered the modules together utilizing the eigengenes.

### Constructing a Prognostic Risk Model on the Basis of Subtype Differential Expression and Co-expressed Genes

To begin with, the 343 samples in the TCGA data set are classified into two groups: the training set as well as the verification set. As a precaution against random allocation variation having an adverse effect on the stability of succeeding modeling, all samples are clustered at random 100 times before being used. In this case, the grouping of samples is conducted in accordance with the ratio of 1:1 for the training set to the verification set. Furthermore, utilizing the training set data, the univariate Cox proportional hazards regression model was performed on differentially co-expressed genes utilizing the survival coxph function in the R-package. The level of *p* < 0.05 was used as the cutoff value for filtering in order to get the genes associated with prognosis.

To significantly reduce the gene range while retaining high accuracy, we performed additional experiments. We employed lasso regression to additionally narrow the prognostic genes, resulting in a reduction in the proportion of genes in the risk model. The Lasso technique is a kind of estimation that uses compression. When it constructs a penalty function, it results in certain coefficients being compressed while others are being set to zero, producing a more refined model. In this way, it keeps its advantages in terms of subset shrinking. It is a skewed estimating method for dealing with complicated collinearity data sets. When assessing variables, it may be used to optimize the selection of parameters, and it could be used to effectively handle the issue of multi-collinearity in regression analysis. The lasso Cox regression analysis was performed utilizing the R software program glmnet (Friedman et al., 2010). In this regard, we investigated the transformation trajectory of every independent variable separately. In addition, we employed a 10-fold cross-validation procedure to construct the model.

### Univariate and Multivariate Cox Analysis and Establishment of a Nomogram

In the TCGA dataset, univariate and multivariable Cox regression analyses were utilized to identify the clinical independence regarding the model. Nomogram is a method that can intuitively and effectively present the data regarding the risk model and may be conveniently applied in predicting the outcomes. The nomogram makes use of the length of the straight line to depict the effect of several factors on the result, as well as the effect of various variable values on the outcomes. To enhance the predictive performance, we integrated multi-factor meaningful variables and established a new nomogram using the Cox model. Additionally, the calibration curve was utilized to determine its predictive performance. Finally, we performed DCA (Decision curve analysis) to determine its credibility.

### Cell Culture

The Chinese Academy of Sciences (Shanghai, China) provided the NCM460 cell line and the human colon cancer cell lines including LoVo, HT29, and HCT116. All the obtained cell lines were grown in RPMI 1640 accompanied by 10 percent fetal bovine serum.

### RNA Isolation and RT-PCR Analysis

The TRIzol reagent (Invitrogen, Carlsbad, CA, United States) was utilized to extract RNA from the tissue samples. Subsequently, QuantiTect Reverse Transcription Kit (Qiagen, Valencia, CA, United States) was employed to convert the isolated RNA into cDNA. SYBR Green (Takara, Otsu, Shiga, Japan) was employed to quantify the results of real-time PCR analyses, and the levels were subsequently standardized to GAPDH levels. The primers used in the upstream and downstream experiments have the following sequences: SPARCL1-forward: 5′-CAA​CTG​CTG​AAA​CGG​TAG​CA-3’; SPARCL1-reverse 5′-GAA​CTC​TTG​CCC​TGT​TCT​GC-3’; HAND1-forward: 5′- AGC​CAC​CAG​CTA​CAT​CGC​CTA​C-3’; HAND1-reverse: 5′- GCGATCCGCCTTCTTGAGTTC-3’;CLEC10A-Forward: 5′- TAC​ACC​TGG​ATG​GGC​CTC​AG -3'; CLEC10A- reverse: 5′- TGT​TCC​ATC​CAC​CCA​CTT​CC -3; PTGS1- forward: 5′- ATC​GCC​ATG​GAA​TTC​AAC​CA-3´; PTGS1- reverse: 5′- GTG​AGC​CCA​CTT​GGA​AGG​AA -3´; PTN- forward: 5′- CCA​TTT​CCC​TTC​CGT​TCC-3'; PTN - reverse: 5′- AGG​TTG​CTA​CCG​CTG​AGT​CC -3´;CXCL1- forward: 5′- CTC​GAG​GCC​CCT​GGG​GCA​GAA​GCC​TC-3´; CXCL1- reverse: 5′- GAT​ATC​GGG​GCT​CAG​CAG​GCG​GGT​CT -3´; CRIP2- forward: 5′- ACT​GAT​GCC​TCC​TCA​CCA​TC-3´; CRIP2- reverse: 5′- TGT​TTG​TGA​GCC​AAC​CAG​AG-3´;

## Results

### Relationship Between Proliferative and Invasive Molecular Subtypes and Prognosis of Colon Cancer

**FIGURE 1:**
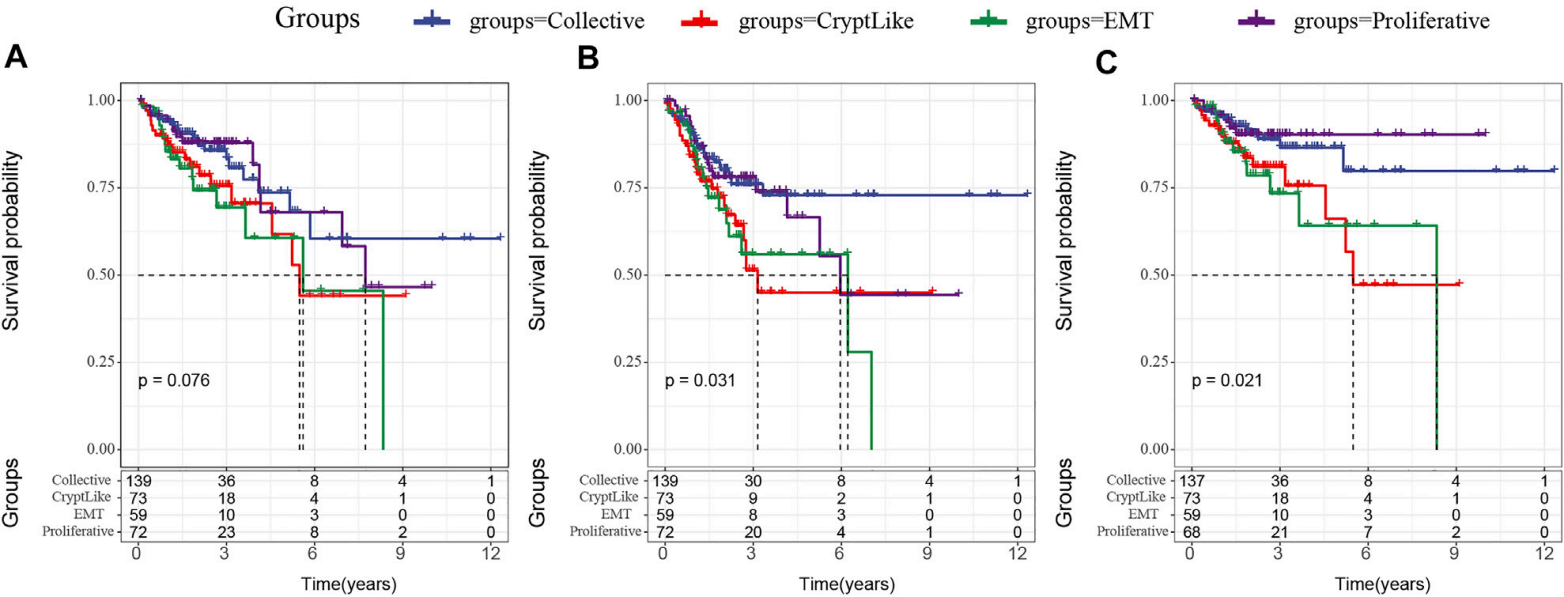
**(A)**: Km curve of the relationship between four subtypes and OS; **(B)**: Km curve of the relationship between four subtypes and PFS; **(C)**: Km curve of the relationship between four subtypes and DSS.

We analyzed the relationship between proliferative and invasive molecular subtypes and patient prognosis. First, we examined the correlation between four molecular subtypes and the OS prognosis. In Figure 1A, it can be observed that there exist marginal considerable differences among the four molecular subtypes (*p* = 0.075). Among the three invasive molecular subtypes, EMT and CryptLike subtypes have the worst prognosis, and Collective has the best prognosis; additionally, we evaluated the correlation between the four subtypes and PFS and found the same findings, that is, EMT and CryptLike subtypes exhibited the most unfavorable prognosis, and Collective exhibited favorable prognosis (Figure 1B); Finally, we analyzed the relationship with DSS. EMT and CryptLike exhibited the most unfavorable prognosis, and Collective exhibited an improved prognosis (Figure 1C). Our results show that there are invasion-related phenotypes in colon cancer, in which EMT and CryptLike are more aggressive.

### Relationship Between Proliferative and Invasive Molecular Subtypes and TMB and Common Gene Variants

**FIGURE 2:**
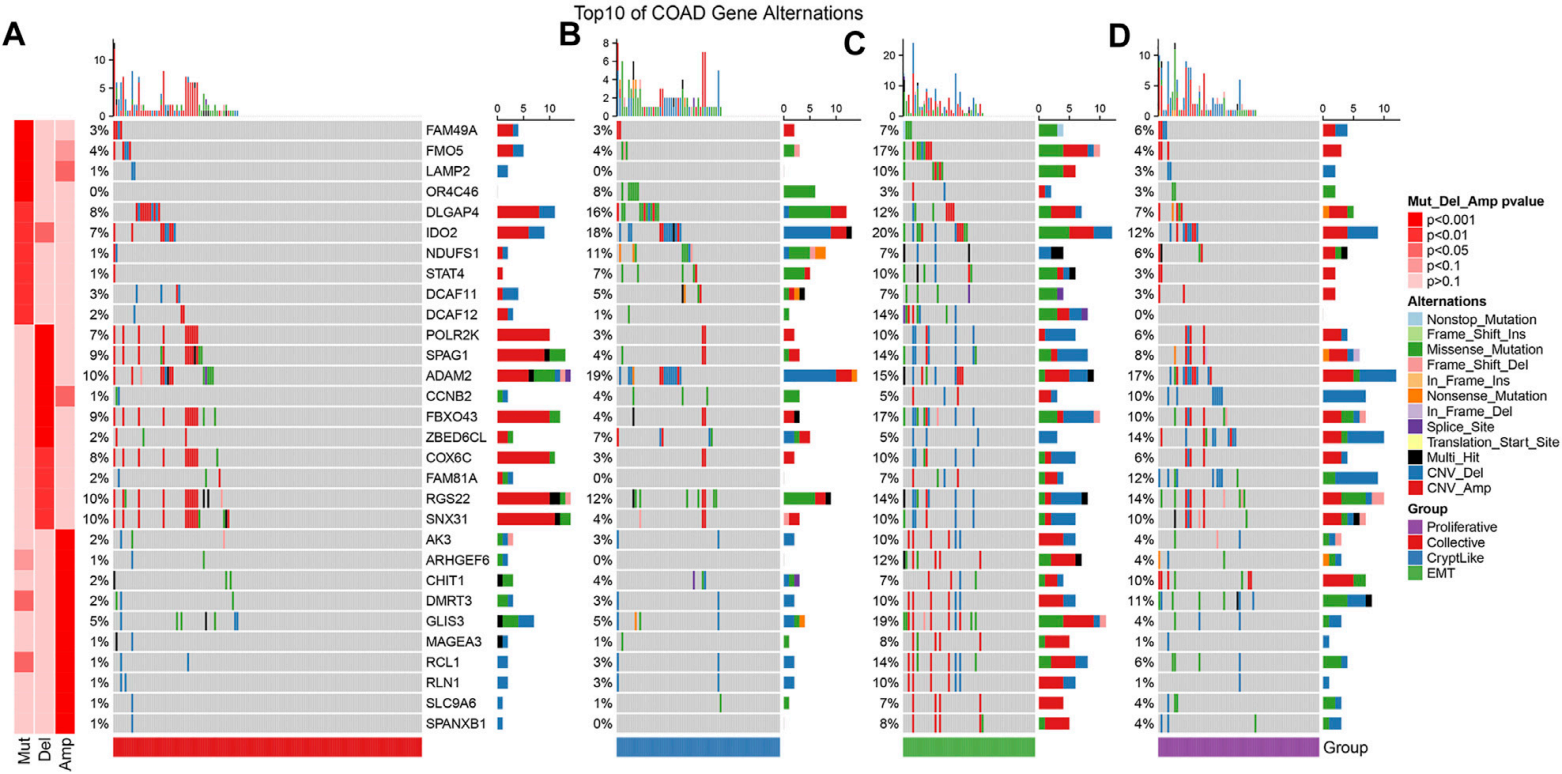
Mutation features of the topmost 10 significantly mutated genes from each subtype sample. **(A–D)** represent collective, cryptlike, EMT, and proliferative subtypes respectively.

We acquired the mutation data set as well as copy number variation data processed by mutect2 software of TCGA-COAD. Then, we used the Fisher test to screen the genes with significant mutation, significant copy deletion, and significant copy amplification differences in each subtype. The mutation characteristics of top10 in each subtype are as shown in Figures 2A–D. It can be observed that the copy number amplification of DCAF12, AK3, ARHGEF6, GLIS3, MAGEA3, RCL1, RLN1, SLC9A6, and SPANXB1 genes in EMT molecular subtypes are substantially elevated in contrast with that in other subtypes.

### Difference in Immune Score Between Proliferative and Invasive Molecular Subtypes

**FIGURE 3:**
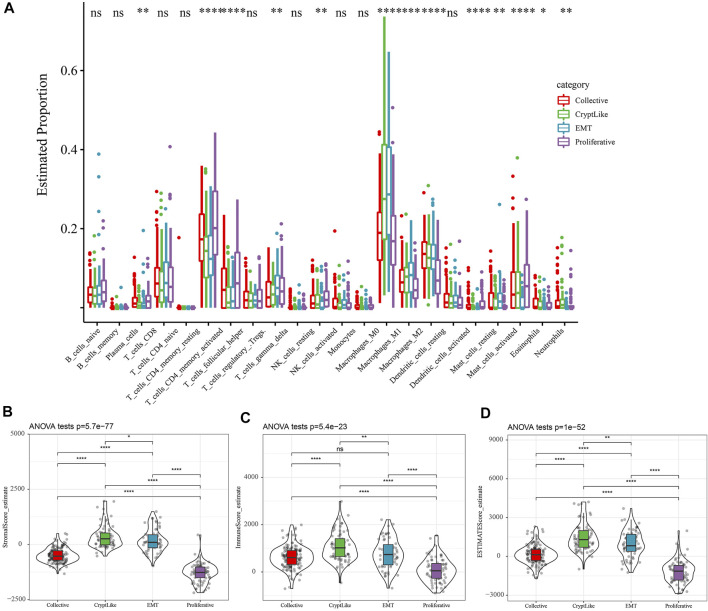
**(A)**: The difference of 22 immune cell components; **(B)**: Differences in matrix scores in different subtypes; **(C)**: The scores of immune infiltration in different subtypes were different; **(D)**: The estimate scores of different subtypes were different.

From the TCGA dataset, the proportion of 22 distinct immune cells in each sample was evaluated by the CIBERSORT method, and the distribution of these immune cell proportions in the four molecular subtypes was as shown in Supplement Figure 1A. The box diagram of immune cells is shown in Figure 3A, and the heat diagram of immune cells is shown in Supplement Figure 1B. It indicated that the Collective, CryptLike, and EMT in macrophases_ M1, Macrophages_ M2 was significantly higher than the proliferative molecular subtype. M1 macrophages play an anti-tumor role by activating the immune system and releasing tumor necrosis factor, nitric oxide, and reactive oxygen species, while macrophages M2 is the main participant and coordinator in promoting tumor progression in the tumor microenvironment(Atri et al., 2018). Immune infiltration analysis showed that CryptLike and EMT molecular subtypes had high immune microenvironment infiltration, and proliferative had the lowest immune microenvironment infiltration. Interestingly, CryptLike and EMT have the highest immune infiltration scores, but the prognosis is the worst, shown in Figures 3B–D.

### Differential Analysis of Proliferative and Invasive Molecular Subtypes in the Efficacy of Immunotherapy/Chemotherapy

**FIGURE 4:**
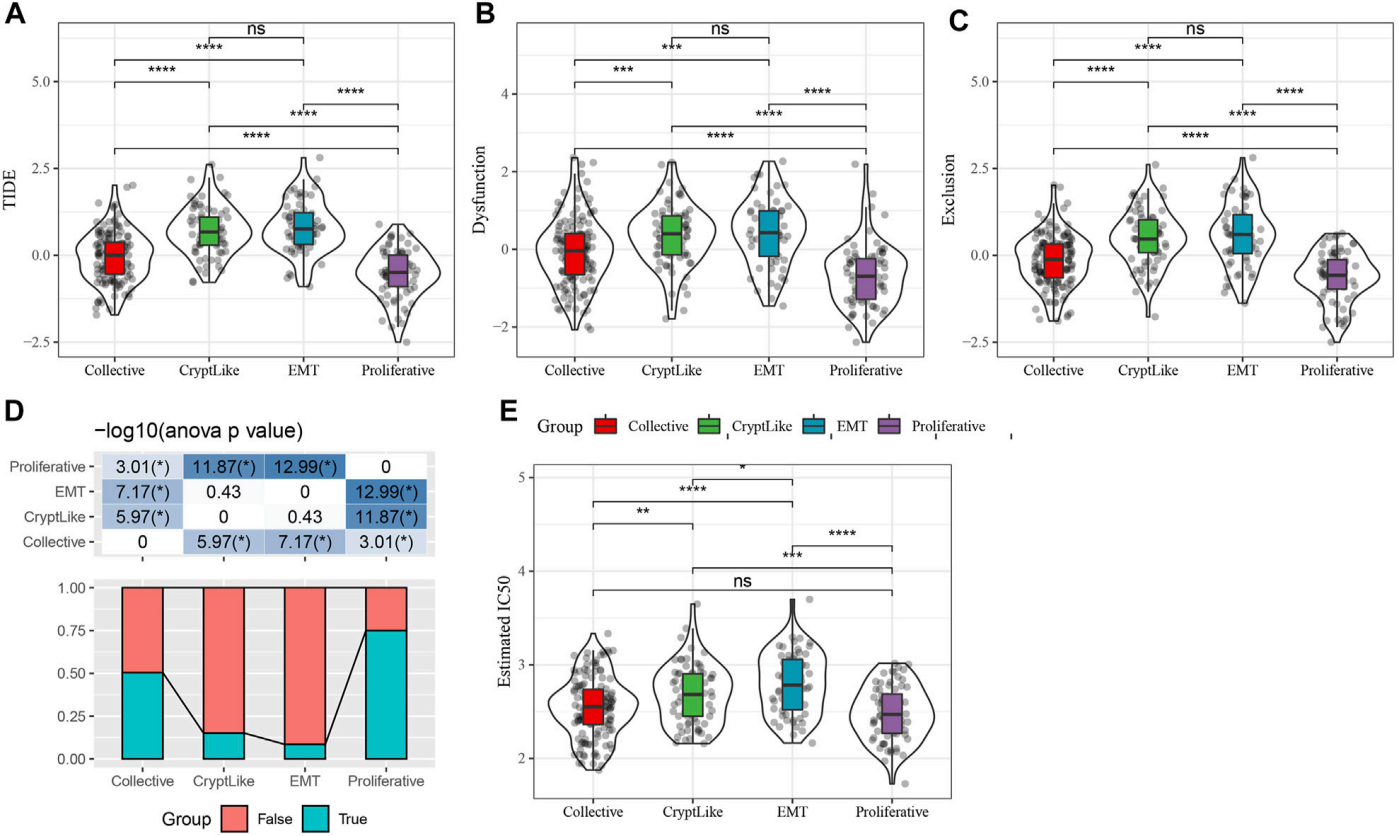
**(A)**: The tide scores of different molecular subtypes were different; **(B)**: T cell dysfunction scores of different molecular subtypes were different; **(C)**: The T cell rejection scores of different molecular subtypes were different; **(D)**: The difference of immune response of different molecular subtypes; **(E)**. The box plots represent the predicted IC50 inhibition concentration for Cisplatin.

We use TIDE (http://tide.dfci.harvard.edu/) Software to evaluate the potential clinical effects of immunotherapy in proliferative and invasive molecular subtypes. The higher the TIDE prediction score, the higher the possibility of immune escape, suggesting that patients are less likely to benefit from immunotherapy. As illustrated in Figure 4A, we discovered that there were substantial differences in TIDE scores between distinct molecular subtypes. The TIDE score of the proliferative subtype is the lowest, which may benefit more from immunotherapy. At the same time, we also compared the predicted T cell rejection score and T cell dysfunction score in different molecular subtypes. As depicted in Figures 4B,C. it is evident that CryptLike and EMT have elevated T cell rejection scores and T cell dysfunction scores than collective and proliferative. It may be that CryptLike and EMT have high immune infiltration scores, but there are T cell dysfunction and T cell rejection, resulting in a poor prognosis of CryptLike and EMT. The distribution difference of different response states to immunotherapy predicted by tide software in different immune molecular subtypes is illustrated in Figure 4D. We discovered that the proportion of response to immunotherapy in collective and proliferative subtypes is substantially elevated as opposed to that in CryptLike and EMT subtypes. In addition, we evaluated the response of distinct subtypes to conventional chemotherapeutic drugs 5-FU and cisplatin. It was found that Collective and Proliferative subtypes were more sensitive to cisplatin than other subtypes (Figure 4E).

### Analysis of Differentially Expressed Genes Among Subtypes

**FIGURE 5:**
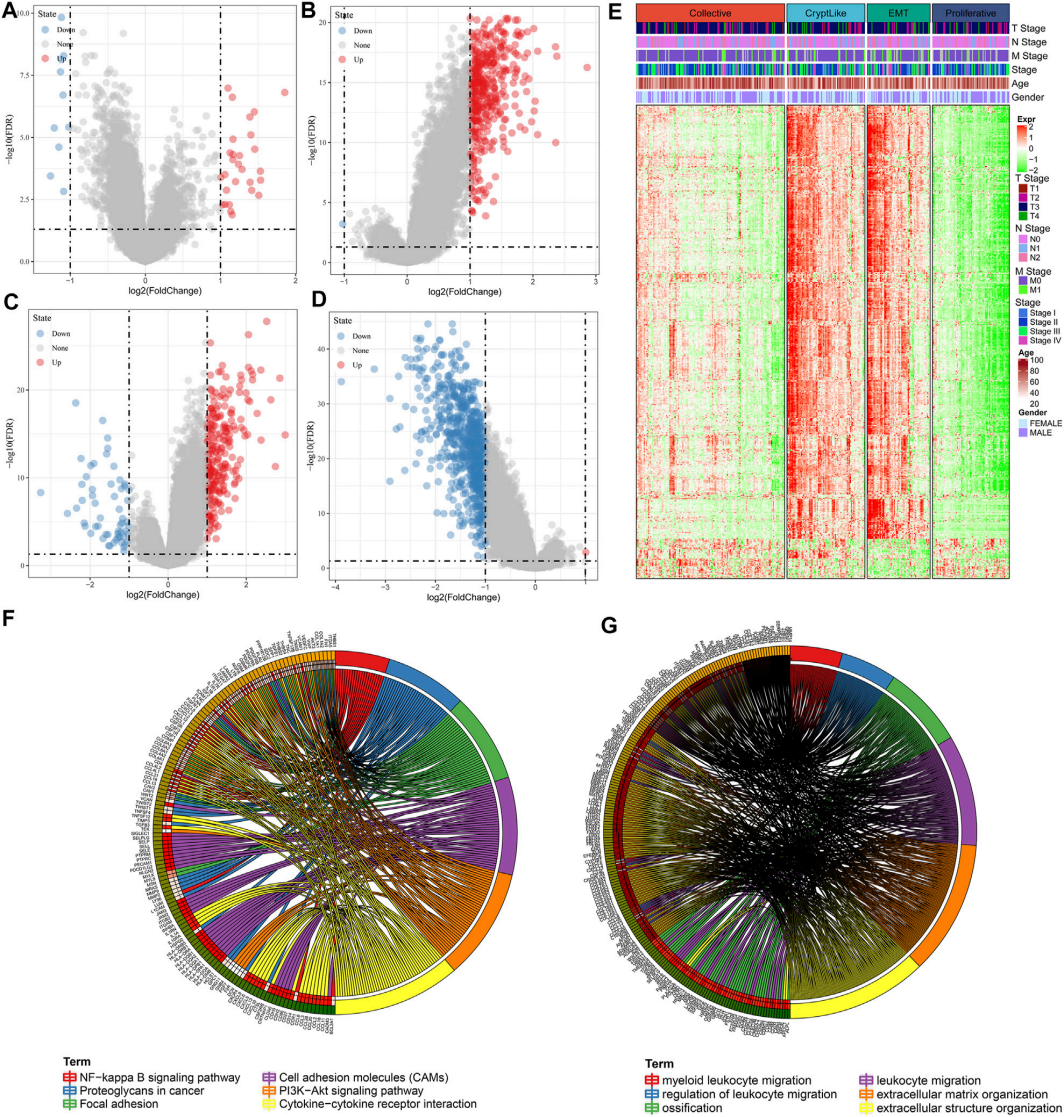
**(A)**: Differences in gene expression between Collective and non-Collective populations are depicted on a volcano map; **(B)**: CryptLike and non-CryptLike groups were compared using a volcano map to identify genes that differed; **(C)**: Volcano map of differential genes between EMT and non-EMT groups; **(D)**: Volcanic map of differential genes between Proliferative and non-Proliferative groups. **(E)**. Heat map of DEGs among subtypes; **(F)**: The results of KEGG enrichment of 846 differential genes; **(G)**: GO-BP enrichment results of 846 different genes, where distinct colors denote distinct pathways, and connecting lines denote genes correlated with pathways.

The DEGs among Collective ∼ Other, CryptLike ∼ Other, EMT ∼ Other. and Proliferative-Other were obtained utilizing the limma package. Filtering was carried out according to the thresholds of | log2fc | > 1 and FDR <0.05. Collective ∼ Other up-regulated and down-regulated DEGs are illustrated as the volcano figure as shown in Figure 5A, which include 11 down-regulated and 28 up-regulated genes, indicating that Collective ∼ Other is mainly up-regulated; CryptLike ∼ Other up-regulated and down regulated DEGs are illustrated as the volcano figure as shown in Figure 5B, which include one down-regulated gene and 394 up-regulated genes, indicating that CryptLike ∼ Other is mainly up-regulated; EMT ∼ Other down-regulated and up-regulated DEGs are illustrated as the volcano figure as shown in Figure 5C, which include 58 down- regulated genes and 232 up- regulated genes. The results show that EMT ∼ Other is mainly up-regulated; The down- regulated and up- regulated DEGs of Proliferative-Other are shown in Figure 5D, including 730 down- regulated genes and one up- regulated gene. The results show that Proliferative-Other is mainly down-regulated. The heat map of DEGs among subtypes is shown in Figure 5E. Furthermore, we performed functional enrichment analysis on 846 DEGs after eliminating redundancy of differential genes among the four subtypes via the clusterProfiler function in R software, and defined the cutoff value of FDR <0.05. The KEGG is enriched in 55 pathways, mainly Focal adhesion, Cytokine-cytokine receptor interaction, PI3K-Akt signaling pathway, Cell adhesion molecules (CAMs), NF-kappa B signaling pathway, Proteoglycans in cancer, and other pathways associated with tumor occurrence and progression as depicted in Figure 5F; There are 1165 GO-BP enrichment results, of which the first six GO-BP are shown in Figure 5G.

### Identify Phenotype Related Coexpression Gene Modules

**FIGURE 6:**
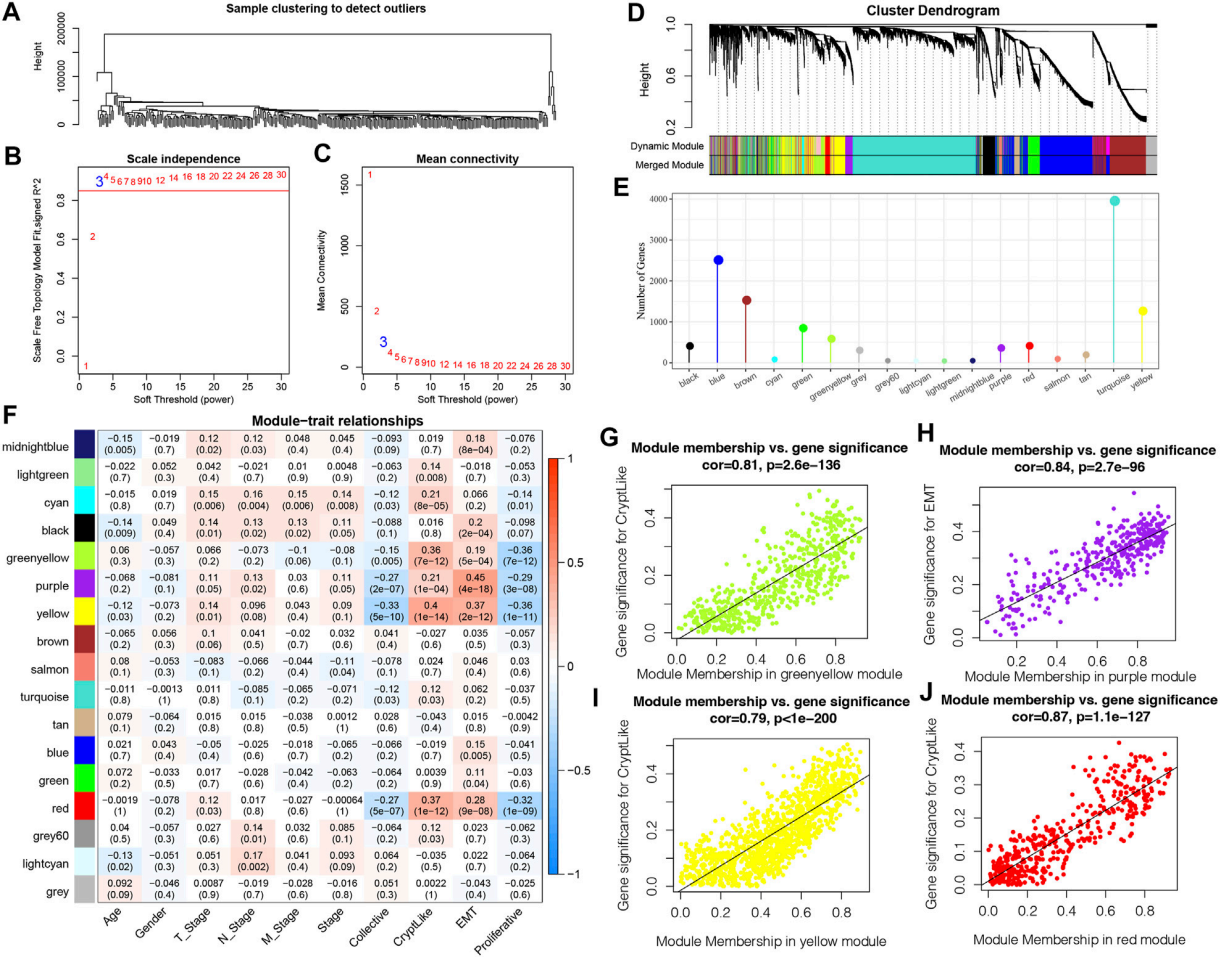
**(A)**: Clustering tree of each sample; **(B)**: An examination of the scale-free fit index for a variety of soft-threshold values (β). **(C)**: For different soft-threshold values, an analysis of the mean connectedness was carried out. **(D)**: Dendrogram illustrating all DEGs/lncRNAs clustered according to a dissimilarity measure (1-TOM); **(E)**: Statistics of the proportion of each module genes; **(F)**: Relationship between clinical information and each module; **(G)**: In the green-yellow module, a scatter plot showing the relationship between module membership and gene value for CryptLike is shown; **(H)**: In the purple module, the scatter graphic shows the relationship between module membership and gene importance for EMT; **(I)**: In the yellow module, a scatter plot showing the relationship between module membership and gene importance for CryptLike is presented; **(J)**: For CryptLike in the red module, the scatter graphic shows the relationship between module membership and gene importance.

We employed the WGCNA module in R software to identify phenotype-related coexpression modules. Specifically, we selected TCGA expression profile data set and screened genes that had MAD of over 50 percent as gene expression profiles. Firstly, we clustered the samples and chose the soft threshold of 3 (Figure 6A). To guarantee a scale-free network, we choose β = 3(Figures 6B–C). Next, the adjacency matrix was generated by transforming the expression matrix. The adjacency matrix was subsequently turned into a topological matrix. To determine the gene module, we first computed the eigenvectors for all modules one by one. Then, we clustered the modules and merged those that are near to one another into newer modules. We next specified the minModuleSize = 30, DeepSplit = 2, and height = 0.25 parameters for the gene module. Finally, a sum of 17 modules was obtained (Figure 6D). Notably, however, the grey module is comprised of a gene set that cannot be combined with other modules. Figure 6E depicts the number of transcripts produced from each module, in which the grey module is a gene module that cannot be allocated. We examined the association between each module and the patients’ M stage, gender, N stage, T stage, age, Stage, and proliferative and invasive molecular subtypes, as illustrated in Figure 6F. It is evident that greenyellow, purple, yellow and red modules have a substantial positive association with CryptLike and EMT, and an inverse association with collective and proliferative. The results of correlation analysis between GS and MM of genes in the module are shown in Figures 6G–J the results show that GS and MM of green, yellow, purple, yellow, and red modules are highly positively correlated.

### Developing a Prognostic Risk Model According to Subtype Differential Expression and Co-expression Genes

#### Construction of Training Set Sample Risk Model

The 343 samples from the TCGA data set have been classified into two categories: the training set as well as the verification set. As a precaution against random allocation bias having an adverse effect on the stability of succeeding modeling, all samples were clustered at random 100 times before being used. To group the samples, a ratio of 1:1 was utilized for the training set to the verification set. The final training set comprises 171 samples, while the test set contains 172 samples.

Further, using the training set data, for the differentially co-expressed genes (399 in total) and survival data, the survival coxph function of the R-package was utilized to carry out a univariate Cox proportional hazards regression model. The filtration level was set at *p* < 0.05. Finally, there were 46 genes related to prognosis.

**FIGURE 7:**
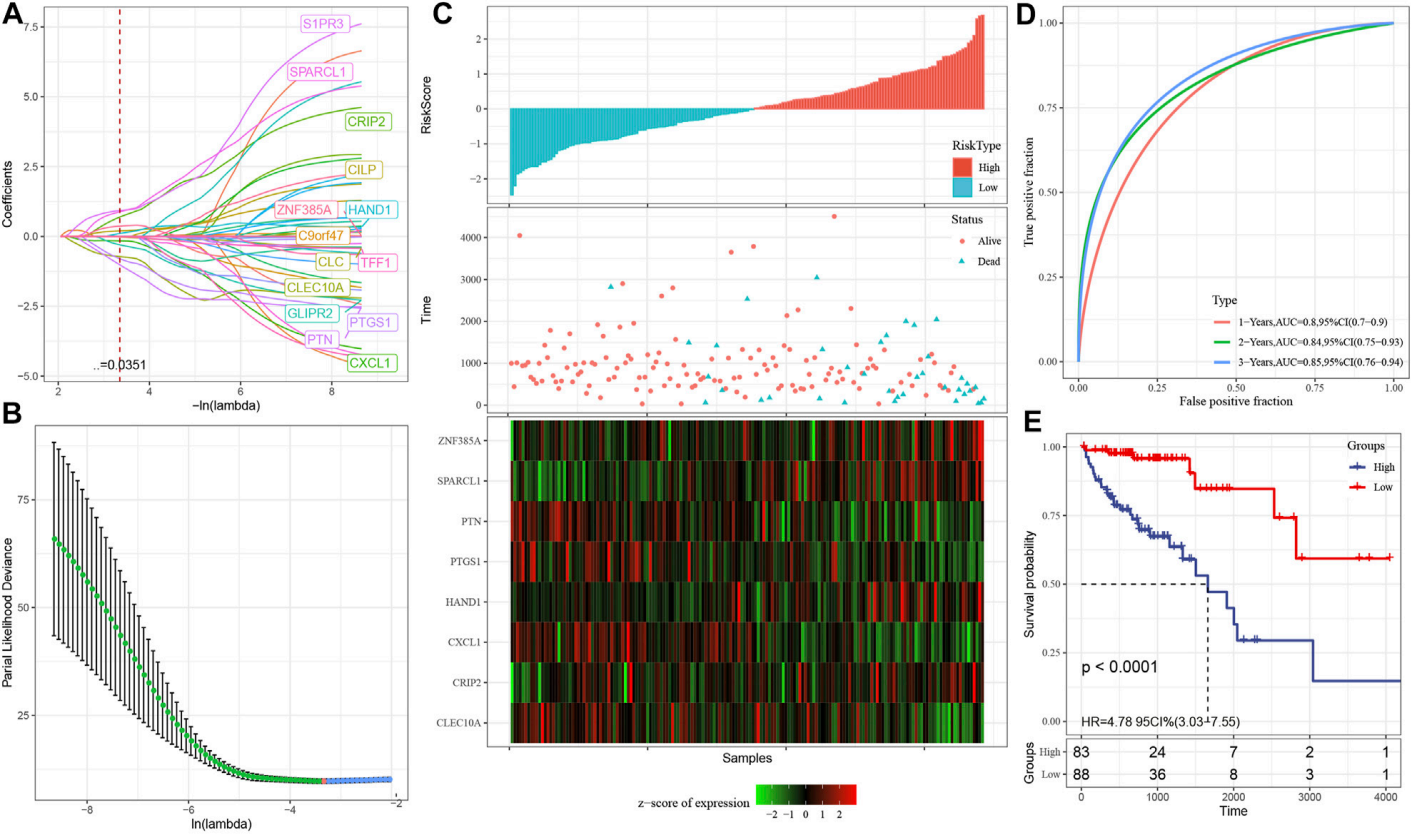
**(A)**: The change trajectory of each independent variable, where the independent variable lambda log value is represented by the horizontal axis, while the independent variable coefficient is represented by the vertical axis; **(B)**: In each lambda, the confidence interval is calculated; **(C)**: The survival status, survival duration, risk score, and eight-gene expression in the TCGA training set; **(D)**: The classification of the ROC curve and AUC is performed based on an 8-gene signature; **(E)**: In the training set, the survival curve distribution of an 8-gene signature was calculated using the KM algorithm.

At present, 46 differentially co-expressed genes related to prognosis have been identified. Nevertheless, because of the substantial proportion of these genes and the inconducive impact on clinical detection, we must additionally reduce the gene range while retaining high accuracy in the process. We further compressed these 46 genes utilizing lasso regression to minimize the proportion of the risk model genes. For the lasso Cox regression analysis, we utilized the glmnet package of R software. As illustrated in Figure 7A, we examined the change trajectory of each independent variable. It is evident that when lambda is progressively increased, the proportion of independent variable coefficients that are on the verge of zero grows progressively as well. To construct the model and examine the confidence interval within each lambda, we employed a 10-fold cross-validation procedure, as depicted in Figure 7B. It can be seen from the figure that when lambda = 0.03507542, the model reaches the optimum. In view of this, in the following stage, we picked 14 genes when lambda = 0.03507542, which were then utilized as targets for the subsequent step.

As part of its stepwise regression procedure, the AIC data criterion is utilized, which takes into account both the statistical fit as well as the total number of components employed for fitting. AIC is reduced using a stepping technique in the Stats package. We begin with the most complicated model and remove one variable at a time to lower AIC. The model performs better when the value is lower than a certain threshold, demonstrating that the model may get a satisfactory fit using fewer features. With the help of this algorithm, we were able to reduce 14 genes to only eight genes: SPARCL1, CXCL1, HAND1, CRIP2, CLEC10A, PTGS1, PTN, and ZNF385A.

Eventually, the signature equation consists of eight genes and is illustrated below: risk score = SPARCL1*1.8291043-CXCL1*0.4539938 + HAND1*0.4792704 + CRIP2*1.5098001-CLEC10A*1.2404183-PTGS1*1.2728945-PTN*2.2581275 + ZNF385A*0.6856336.

As illustrated in Figure 7C, we computed the risk score of each sample based on the level of expression of the sample and plotted the risk score distribution for the sample. From the figure, it is evident that the mortality rate for samples having an elevated risk score was substantially greater in contrast with the death rate for samples having a reduced risk score, indicating that high-risk score samples have a poorer prognosis. The expression of eight separate signature genes differs in response to an increase in risk value. The elevated expression of SPARCL1, HAND1, CRIP2 and ZNF385A was identified as risk variables. The high expression of CXCL1, CLEC10A, PTGS1 and PTN were correlated with low risk, indicating that these genes have a protective function. In addition, we utilized the timeROC function of the R software to examine the ROC of the prognostic categorization of risk score. We examined the categorization effectiveness of prognostic predictions in three different time periods: one-, two-, and three-year (Figure 7D). It is evident that the model has a large area underneath the AUC line; Eventually, we computed the zscore on the risk scores and classified the samples with risk scores larger than zero into two groups: high- and low-risk groups. After plotting the KM curve as illustrated in Figure 7E, we identified a substantial difference between the two groups (*p* < 0.0001), of which 88 samples were categorized into the low-risk group while 83 samples were categorized into the high-risk group.

#### Multiple Data Sets Confirm the Stability of the Eight-Gene Signature

**FIGURE 8:**
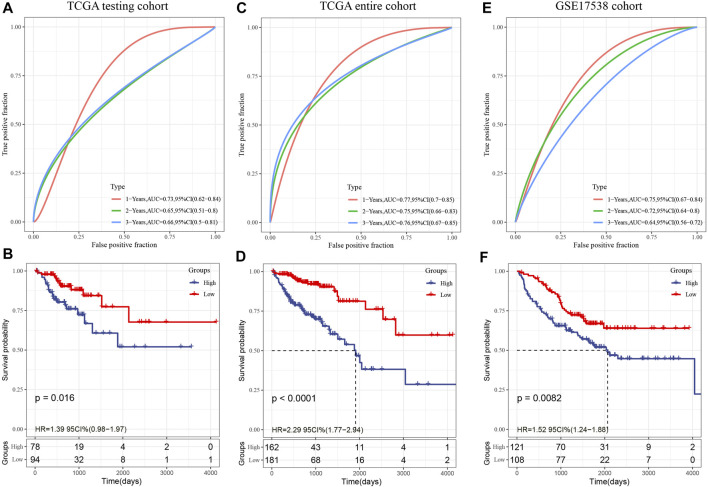
**(A)**: In the TCGA test set, the ROC curve and AUC were categorized utilizing an 8-gene signature; **(B)**: In the TCGA test set, the KM survival curve distribution of an eight-gene signature was examined; **(C)**: In the whole TCGA data set, the ROC curve and AUC were categorized utilizing an 8-gene signature; **(D)**: The distribution of the KM survival curve for an eight-gene signature throughout the whole TCGA data set. **(E)**: The classification of the ROC curve and AUC is based on an eight-gene signature; **(F)**: The KM survival curve illustrating the distribution of the eight-gene signature within the independent validation data set GSE17538.

The validation set from TCGA as well as all other data sets were utilized to assess the robustness of the model, with a similar model and equivalent coefficients as the training set being used to make the determination. After determining the levels of expression of the risk score of each sample, the ROC analysis of the risk score of each sample was performed utilizing the timeROC function in the R software. We examined the categorization effectiveness of prognostic predictions in three different time periods: one-, two-, and three-year. We subsequently computed the zscore and the risk scores, classified the samples with risk scores larger than zero into two groups: high-and low-risk. Then, we created the KM curve. In the TCGA internal validation data, as depicted in Figures 8A–B, we found that the model exhibits an elevated AUC in the independent internal validation data set, and a substantial difference was observed between the low- and high-risk groups according to the risk value (*p* = 0.016). Specifically, 94 samples were categorized into the low-risk group while 78 samples were categorized into the high-risk group. In the whole TCGA data set, as depicted in Figures 8C,D, it was discovered that the AUC of the model in 3 years reaches 0.76, and there is also a considerable difference between the low- and high-risk groups according to the risk value of the samples (*p* < 0.0001), of which 162 samples are categorized into the high-risk groups while 181 samples fall under the low-risk group.

In the external verification set GSE17538, we employed a similar model and equivalent coefficients as those utilized in the training set. In a similar manner, the risk score of each sample was determined in accordance with the degree of expression of the sample. In addition, we utilized the timeROC function in the R software to investigate the ROC of the prognostic categorization of the risk score. We examined the categorization effectiveness of prognostic predictions in three different time periods: one-, two-, and three-year (Figure 8E); In the end, we calculated the risk score, classified the samples into low- and high-risk groups, and plotted the KM curve, as demonstrated in Figure 8F. The findings illustrated a statistically substantial difference between the two groups (*p* = 0.0082), of which 121 samples are categorized into the high-risk group while 208 samples fall under the low-risk group.

#### Cox Analysis and Nomogram Construction of 8-Gene Signature

**FIGURE 9:**
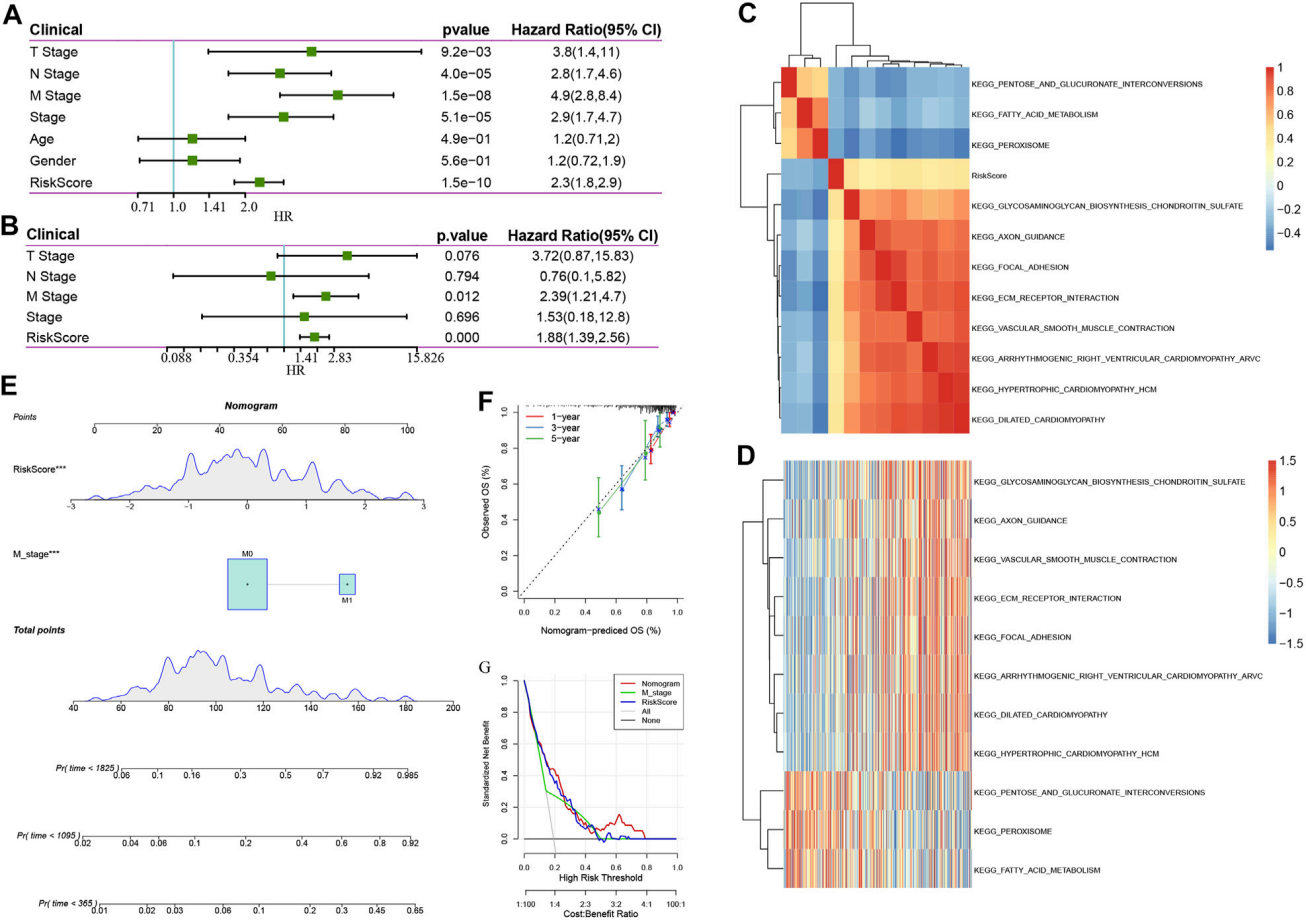
**(A)**: Findings of the univariate analysis and clinical characteristics of the risk score; **(B)**: Findings of the multivariate analysis and clinical characteristics of the risk score. **(C)**. KEGG pathways that are substantially linked to risk score are grouped together in a cluster; **(D)**: Changes in the association between KEGG pathways that are strongly associated with the risk score and ssGSEA score (the sample is represented by the abscissa, and the risk scores are elevated from the left side to the right side). **(E)**: Nomogram model of M stage and risk score combination; **(F)**: One-, three-, and five-year Calibration curve of the nomogram; **(G)**: Decision curve of M stage, risk score, and nomogram.

The risk score was shown to be strongly linked to survival in the TCGA data set, according to univariable Cox regression analysis (Figure 9A). Corresponding multivariable Cox regression analysis found that risk score was still substantially linked to survival (Figure 9B HR = 1.86, 95% CI = 1.42—2.45, P < 1 e-5). The findings described above demonstrate that our eight-gene signature model exhibits strong predictive performance in terms of clinical applicability. Specifically, to examine the correlation between biological activities and risk scores among various samples, we utilized the gene expression profiles related to these samples, performed single sample GSEA analysis utilizing the GSVA package of the R software, measured the scores of each sample on various functions, and acquired the ssGSEA scores of each sample related to each function, and thereafter obtained the connection between any of these functions and risk scores. Figure 9C illustrates our selection of functions with correlations larger than 0.3. It could be observed that eight pathways were positively linked to the sample risk score, while three pathways were inversely linked to the sample risk score. As demonstrated in Figure 9D, we chose the 11 topmost correlated KEGG pathways and performed cluster analysis on them based on their enrichment ratings. We found that the tumor-related pathways such as KEGG_VASCULAR_SMOOTH_MUSCLE_CONTRACTION, KEGG_FOCAL_ADHESION, and KEGG_ECM_RECEPTOR_INTERACTION increased as the risk score continued to increase.

The univariate and multivariate analyses illustrated that in addition to the risk score, the clinical feature M stage was found to independently function as a prognostic marker, implying that they had complementary value. To additionally enhance the prediction performance, we integrated the M stage and risk score, established a new nomogram using the Cox model, and combined the two independent prognostic predictors (Figure 9E), according to this model, we found that risk score has the most contribution to OS, with the M stage coming second. In addition, we utilized the calibration curve to determine the predictive accuracy regarding the model (Figure 9F). The findings illustrated that the predictive calibration curve of three estimation points in one-, three-, and five-year is proximate to the standard curve, indicating that the model possesses strong predictive accuracy. Furthermore, we utilized DCA (decision curve) to assess the reliability (Figure 9G) of the model. It was discovered that at a high-risk threshold of 0.1–0.3, the advantages of nomogram and risk score substantially outweigh those of M stage and extreme curve by a wide margin. When the high-risk threshold is 0.3–0.5, the benefits of risk score, nomogram, and M stage are similar. When the high-risk threshold is greater than 0.5, the net benefits of the nomogram are greater than 0, which has clinical significance. On the whole, risk score and nomogram have good reliability when the high-risk threshold is 0.1–0.5. When the high-risk threshold is greater than 0.5, the nomogram has a high benefit rate.

### Comparison Between Risk Model and Other Models

**FIGURE 10:**
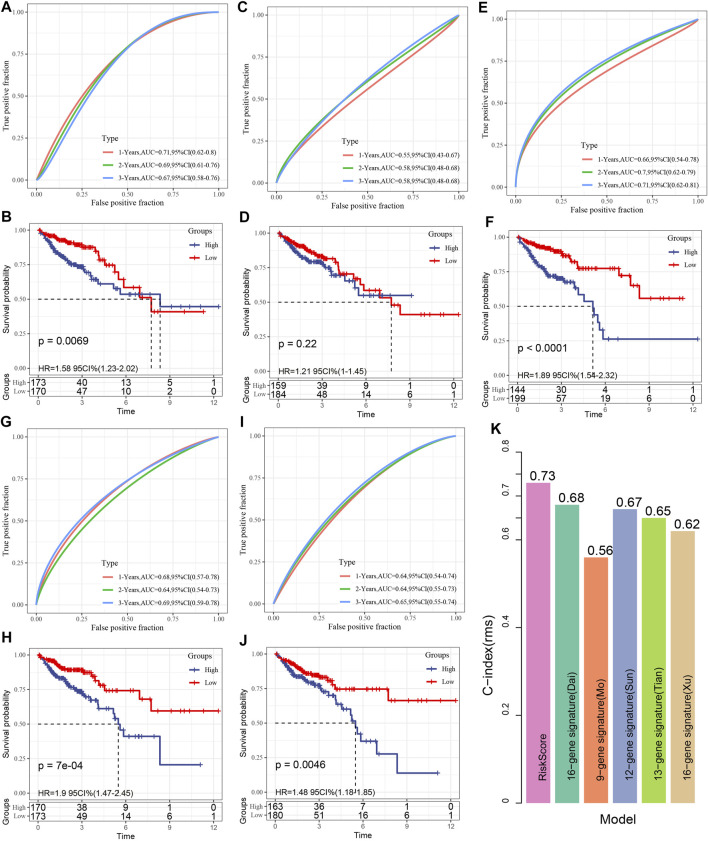
**(A, B)**: The receiver operating characteristic (ROC) of the 15 gene signature (Dai) risk model, as well as the COAD KM curve of low- and high-risk clustered samples; **(C, D)**: The ROC curve of the 9-gene signature (Mo) and the COAD KM curve of the low/high; **(E, F)**: The ROC of 12-gene signature(Sun) as well as COAD KM curve of Low/High; **(G, H)**: ROC of 13-gene signature(Tian) and COAD KM curve of Low/High; **(I, J)**: ROC of 15-gene signature(Xu) and COAD KM curve of Low/High/; **(K)**. C-indexes of six prognostic risk models.

Following a review of the literature, we eventually obtained five prognosis-related risk models to be used for comparison with our eight-gene model including 15-gene signature (Dai) (Dai et al., 2018), 9-gene signature(Mo) (Mo et al., 2019), 12-gene signature(Sun) (Sun et al., 2018), 13-gene signature(Tian) (Tian et al., 2017),15-gene signature(Xu) (Xu et al., 2017). In the attempt of making the model more similar to some degree, the risk score of each COAD sample from TCGA was computed as per the matching genes in the five models, utilizing the same approach, and the risk score was zscore. After the zscore, the samples whose risk score was found to exceed zero were categorized into low- and high-risk groups. The difference in COAD prognosis between the two groups was computed and compared. Figures 10A–J depicts the ROC and COAD-KM curves of the five models. It can be seen that the AUC values of the five models in 1, 2, and 3 years are lower than our model. The COAD prognosis of the low and high grouped samples of the five models except for the 9-gene signature (Mo) model was different (log-rank *p* < 0.05). Our model was discovered to be more rational and accurate even when fewer genes are used. Moreover, we also utilized the “rms” package in R to determine the concordance index (C-index) of various models in order to compare the predictive accuracy among models. According to Figure 10K, the C-index of the eight-genes model is the greatest, showing that the overall performance of our model is better than the other five models.

### Verification of 8 Gene Expression

**FIGURE 11:**
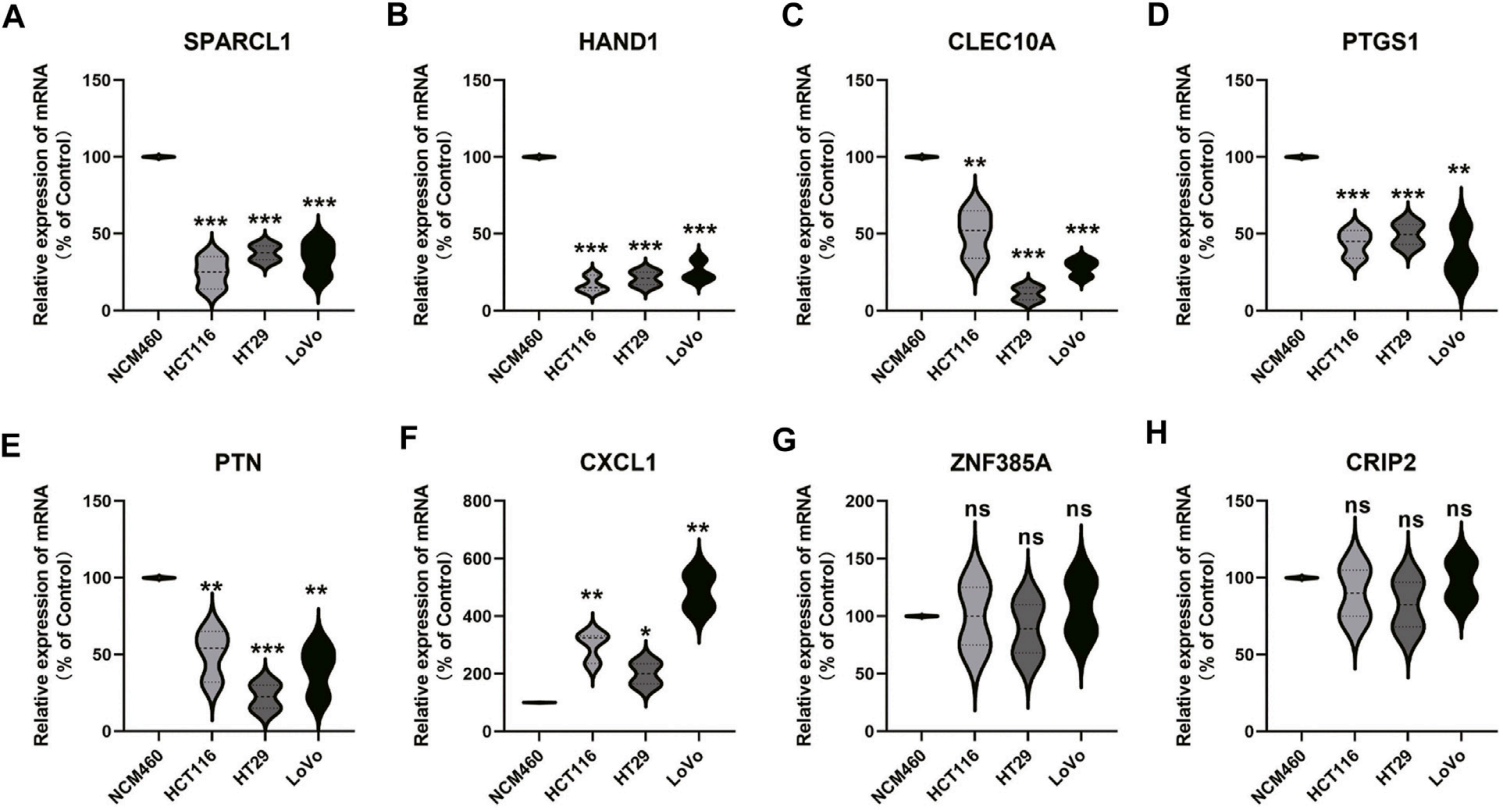
Gene expression verification in normal samples and tumor samples. From **(A to H)** represent SPARCL1, HAND1, CLEC10A, PTGS1, PTN, CXCL1, ZNF385A, and CRIP2, respectively.

In order to study the expression patterns of genes in tumors and normal cell lines, PCR was used to detect the mRNA expression of genes, and the results showed that the expression of SPARCL1, HAND1, CLEC10A, PTGS1 genes in tumor group was significantly lower than that in the normal group. However, the expression of CXCL1 in tumor group was significantly higher than that in the control group. There is no difference in the expression of ZNF385A and CRIP2 between the tumor and the control group (Figures 11A–H).

## Discussion

The tumor microenvironment performs an integral function in the progression of tumors and it is not only a barrier for tumor cell metastasis (Wang and Hielscher, 2017; Lin et al., 2019), but also a favorable “soil” for tumor cell reproduction, and endows tumor cells with the ability to metastasize to a distance (Mao and Schwarzbauer, 2005). In addition to tumor cells, the tumor microenvironment is composed of stromal cells, inflammatory cells, vascular system, and extracellular matrix (Frankel et al., 2017), in which tumor-related immune cells are an important part. Previous studies have shown that stromal-rich tumors have been proved to be unfavorable for prognosis, and the same conclusion has been reached in colorectal cancer (West et al., 2010; van Pelt et al., 2018; Geessink et al., 2019), which is consistent with our results in this study. The EMT and cryptlike subtypes with the worst prognosis have high immune microenvironment infiltration. Of course, our results also provide another possible reason. In these two subtypes, the T cell rejection score and T cell dysfunction score are high. It is speculated that the genetic abnormalities of tumor cells will reprogram the surrounding infiltrating cells, resulting in the tumor microenvironment conducive to tumor development. This gene abnormality is usually manifested in the abnormal amplification of some specific genes. These results show that COAD leads to different prognostic outcomes due to its high heterogeneity. At present, the clinical TNM stage may not fully reflect its prognosis and the choice of treatment methods. The 2020 ESMO colon cancer clinical practice guide recommends the application of immune scores to predict the prognosis of patients with colon cancer and guide clinical medication (Argilés et al., 2020). On the other hand, our results show that the proportion of response to immunotherapy in Collective and Proliferative subtypes is substantially elevated as opposed to that in CryptLike and EMT subtypes, which cannot be perfectly explained by the results of this study. However, we speculate that this may be related to the imbalance of intestinal-specific microbiota. Studies have shown that in crypt-like infiltration, mucosal microbiota still exists even if the tumor has distant metastasis. While in EMT invasion, the tumor seems to lose the mucosal microbiota (Bullman et al., 2017). The state of intestinal flora also performs an integral function in the efficacy of immune checkpoint inhibitors (Westdorp et al., 2021). Unfortunately, we lack the analysis of intestinal flora in this study. We will improve this part of data in further research to provide a basis for further guiding clinical medication.

Proliferation and metastasis are the two characteristics of tumors. There is a deviation in the gene expression of these two characteristics of tumors. We extracted the DEGs of these two groups of COAD to screen the genes that perform an instrumental function in tumors. The results of pathway enrichment suggest that the pathways involved by these genes are concentrated in Cell adhesion molecules (CAMs) (Bronikowska et al., 2021), cytokine receptor interaction (Wen et al., 2021), focal adhesion (Lu et al., 2021), NF kappa B signaling pathway (Takakura et al., 2021), proteoglycans in cancer (Jinmin Sun et al., 2021), PI3K Akt signaling pathway (Xue et al., 2021), etc, which are strongly associated with to the onset and progression of tumors. Further cluster analysis showed that CryptLike, EMT, Collective and Proliferative were distributed in different modules respectively. These results showed that different clinical subtypes of colorectal cancer not only showed differences in tumor behavior, but also the fundamental reason was the abnormal activation of different genes and pathways, which was not only an important reason for tumor progression but also a key clinical target.

In order to further enhance clinical applicability, we established prognostic gene signatures related to differential genes. After lasso regression analysis, the combination with the maximum occurrence frequency includes 8 genes: SPARCL1 (SPARC-like protein 1), CXCL1 (Growth-regulated alpha protein), HAND1 (Heart- and neural crest derivatives-expressed protein 1), CRIP2 (Cysteine-rich protein 2), CLEC10A(C-type lectin domain family 10, member A), PTGS1 (Prostaglandin G/H synthase 1), PTN (Pleiotrophin), ZNF385A (Zinc finger protein 385A), The role of these genes in tumors has been preliminarily studied. SPARCL1 is considered a tumor suppressor, which inhibits tumor progression in a variety of tumors. SPARCL1 may prevent the activity of colorectal cancer through its DNA methylation (Hu et al., 2021). The expression of SPARCL1 in HeLa cells is low at the protein level and transcription level. When SPARCL1 is overexpressed in HeLa cells the proliferation, migration, and invasion of cells are strongly inhibited (Zhang et al., 2021). CXCL1, as a chemokine, has been proved to promote tumor progression in a variety of tumors. The CXCL1 overexpression has a positive relationship with the migration and invasive activity of osteosarcoma cell lines (Lee et al., 2021). In acute leukemia, bladder cancer, and other tumors, its expression is increased, and its prognosis is poor, (Xiaoqi Sun et al., 2021; Yazdani et al., 2021). HAND1 is a mesodermal marker (Yi et al., 2020). At present, there are many studies on its role in EMT, but there are contradictions about its role in tumors. HAND1 is expressed in invasive gastrointestinal stromal tumors (GIST) (Hemming et al., 2021). However, in medulloblastoma, HAND1 expression may be the key to weaken EMT, which may be different from previous research conclusions (Asuthkar et al., 2016), but it may be related to different apparent modifications of HAND1 in tumors (Tan et al., 2014). CRIP2 is a transcription factor, which is an unfavorable prognostic factor for breast cancer as well as colorectal cancer (Shi et al., 2016; Zhang et al., 2020). CRIP2 is an autophagy inhibitory protein. CRIP2 mediated copper metabolism activates autophagy in cancer cells (Chen et al., 2021). Moreover, CRIP2 was confirmed to be up-modulated at the mRNA and protein levels of anti-radiation cells, which is a potential diagnostic biomarker and a key biomarker for predicting prognosis (Li et al., 2021). CLEC10A, also known as MGL (macrophage galactose type C lectin), has recently been reported to perform a crucial part in enhancing immune cell activities. CLEC10A can recognize tumor-associated antigens and pass them to CD4 T cells (van Vliet et al., 2007). Moreover, CLEC10A has been shown to greatly enhance the stimulation of antigen-specific CD8 T cells (Napoletano et al., 2012). The role of CLEC10A in enhancing the anticancer effects of immune cells has undoubtedly drawn the interest of researchers, and it has been identified as a potential target for cancer immunotherapy treatment (Eggink et al., 2018). Constitutive cyclooxygenase (COX) - 1 (gene PTGS1) is significantly related to the concentration of PGE2 in the colon and is highly expressed in colon cancer. Therefore, PTGS1 may be regarded as another potential target for colon cancer prevention in high-risk groups (Sidahmed et al., 2016; Ayiomamitis et al., 2019). In colorectal cancer, PTN may play a role as the downstream of PRPH and promote tumor progression (Huang et al., 2021). Tumor-related macrophages increase the proportion of tumor stem cells in lymphoma via the secretion of PTN (Wei et al., 2019). Upregulation of PTN by activating the NF- κ B pathway promotes tumor cell proliferation, inhibits apoptosis and chemosensitivity (Huang et al., 2018). According to the results of a meta-analysis, increased expression of PTN was substantially associated with advanced TNM stage and dismal OS in cancer patients (Zhou et al., 2018). ZNF385A, as RNA binding proteins (RBPs), is a constituent factor of the COAD prognosis model, but its specific mechanism is not clear (Chang et al., 2021). These results show that although these genes play different roles in tumors, according to the current research results, more genes may participate in apparent modification, which may also be why there exist obvious differences in the expression of these genes in distinct phenotypes of COAD, which performs an instrumental function in the prognosis of COAD, but this needs our further verification.

In conclusion, in this study, we further analyzed the differences between molecular subtypes of prognosis of patients with COAD, TMB and common gene variants, immune score, and efficacy of immunotherapy/chemotherapy based on the research of Shayingzhao et al. Moreover, the risk score prognostic model of 8 genes is constructed according to the differential genes. The risk score constructed according to these 8 genes can divide the patients into low- and high-risk groups. The death proportion of the samples with a high-risk score is substantially elevated as opposed to that with the low-risk score, implying that the high risk score samples exhibit unfavorable prognoses. Further application of the training set as well as verification set have higher AUC shows that risk score constructed by our 8 genes has a stable role in predicting prognosis and provides a basis for clinical precision treatment.

## Data Availability

The datasets presented in this study can be found in online repositories. The names of the repository/repositories and accession number(s) can be found in the article/Supplementary Material.

## Supplementary Material

Supplementary Table S1 A total of 366 samples were divided into four subtypes based on TCGA-COAD.
