# Supplementary material for: Based on Molecular Subtypes, Immune Characteristics and Genomic Variation to Constructing and Verifying Multi-Gene Prognostic Characteristics of Colorectal Cancer
Source: Front Cell Dev Biol. 2022 Feb 23;10:828415. doi: 10.3389/fcell.2022.828415 (PMC8905350; doi:10.3389/fcell.2022.828415)
Supplement: Supplementary file 1 [file Table1.DOCX]

Tag,Group

TCGA-5M-AAT5-01,Proliferative

TCGA-A6-3809-01,Proliferative

TCGA-A6-5661-01,Proliferative

TCGA-A6-5665-01,Proliferative

TCGA-A6-5666-01,Proliferative

TCGA-A6-6140-01,Proliferative

TCGA-A6-6650-01,Proliferative

TCGA-A6-6652-01,Proliferative

TCGA-A6-6780-01,Proliferative

TCGA-AA-3488-01,Proliferative

TCGA-AA-3494-01,Proliferative

TCGA-AA-3495-01,Proliferative

TCGA-AA-3664-01,Proliferative

TCGA-AA-3678-01,Proliferative

TCGA-AA-3688-01,Proliferative

TCGA-AA-3819-01,Proliferative

TCGA-AA-3844-01,Proliferative

TCGA-AA-3854-01,Proliferative

TCGA-AA-3979-01,Proliferative

TCGA-AA-A00L-01,Proliferative

TCGA-AA-A00U-01,Proliferative

TCGA-AA-A00W-01,Proliferative

TCGA-AA-A00Z-01,Proliferative

TCGA-AA-A010-01,Proliferative

TCGA-AA-A01F-01,Proliferative

TCGA-AA-A01G-01,Proliferative

TCGA-AA-A01I-01,Proliferative

TCGA-AA-A01Q-01,Proliferative

TCGA-AA-A01T-01,Proliferative

TCGA-AA-A01V-01,Proliferative

TCGA-AA-A01Z-01,Proliferative

TCGA-AA-A02K-01,Proliferative

TCGA-AA-A02Y-01,Proliferative

TCGA-AA-A03F-01,Proliferative

TCGA-AD-6888-01,Proliferative

TCGA-AD-6889-01,Proliferative

TCGA-AD-6963-01,Proliferative

TCGA-AY-5543-01,Proliferative

TCGA-AY-6197-01,Proliferative

TCGA-AY-6386-01,Proliferative

TCGA-AY-A54L-01,Proliferative

TCGA-AY-A69D-01,Proliferative

TCGA-AY-A71X-01,Proliferative

TCGA-AZ-4313-01,Proliferative

TCGA-AZ-6598-01,Proliferative

TCGA-AZ-6606-01,Proliferative

TCGA-AZ-6608-01,Proliferative

TCGA-CK-4952-01,Proliferative

TCGA-CM-4746-01,Proliferative

TCGA-CM-5864-01,Proliferative

TCGA-D5-5540-01,Proliferative

TCGA-D5-6530-01,Proliferative

TCGA-D5-6537-01,Proliferative

TCGA-DM-A1D4-01,Proliferative

TCGA-DM-A1D6-01,Proliferative

TCGA-DM-A288-01,Proliferative

TCGA-DM-A28E-01,Proliferative

TCGA-DM-A28H-01,Proliferative

TCGA-DM-A28K-01,Proliferative

TCGA-DM-A28M-01,Proliferative

TCGA-G4-6295-01,Proliferative

TCGA-G4-6306-01,Proliferative

TCGA-G4-6309-01,Proliferative

TCGA-G4-6317-01,Proliferative

TCGA-G4-6320-01,Proliferative

TCGA-G4-6586-01,Proliferative

TCGA-G4-6626-01,Proliferative

TCGA-NH-A50T-01,Proliferative

TCGA-NH-A6GB-01,Proliferative

TCGA-QG-A5YX-01,Proliferative

TCGA-QG-A5Z2-01,Proliferative

TCGA-QL-A97D-01,Proliferative

TCGA-RU-A8FL-01,Proliferative

TCGA-5M-AATA-01,Collective

TCGA-A6-2672-01,Collective

TCGA-A6-2676-01,Collective

TCGA-A6-2678-01,Collective

TCGA-A6-2680-01,Collective

TCGA-A6-2686-01,Collective

TCGA-A6-3807-01,Collective

TCGA-A6-4105-01,Collective

TCGA-A6-4107-01,Collective

TCGA-A6-5660-01,Collective

TCGA-A6-6137-01,Collective

TCGA-A6-6649-01,Collective

TCGA-A6-6653-01,Collective

TCGA-A6-A565-01,Collective

TCGA-A6-A567-01,Collective

TCGA-AA-3492-01,Collective

TCGA-AA-3506-01,Collective

TCGA-AA-3509-01,Collective

TCGA-AA-3510-01,Collective

TCGA-AA-3511-01,Collective

TCGA-AA-3516-01,Collective

TCGA-AA-3517-01,Collective

TCGA-AA-3518-01,Collective

TCGA-AA-3519-01,Collective

TCGA-AA-3520-01,Collective

TCGA-AA-3521-01,Collective

TCGA-AA-3524-01,Collective

TCGA-AA-3525-01,Collective

TCGA-AA-3526-01,Collective

TCGA-AA-3529-01,Collective

TCGA-AA-3534-01,Collective

TCGA-AA-3538-01,Collective

TCGA-AA-3542-01,Collective

TCGA-AA-3543-01,Collective

TCGA-AA-3544-01,Collective

TCGA-AA-3549-01,Collective

TCGA-AA-3553-01,Collective

TCGA-AA-3555-01,Collective

TCGA-AA-3556-01,Collective

TCGA-AA-3560-01,Collective

TCGA-AA-3561-01,Collective

TCGA-AA-3562-01,Collective

TCGA-AA-3655-01,Collective

TCGA-AA-3660-01,Collective

TCGA-AA-3662-01,Collective

TCGA-AA-3663-01,Collective

TCGA-AA-3666-01,Collective

TCGA-AA-3667-01,Collective

TCGA-AA-3672-01,Collective

TCGA-AA-3673-01,Collective

TCGA-AA-3675-01,Collective

TCGA-AA-3679-01,Collective

TCGA-AA-3681-01,Collective

TCGA-AA-3685-01,Collective

TCGA-AA-3692-01,Collective

TCGA-AA-3697-01,Collective

TCGA-AA-3710-01,Collective

TCGA-AA-3712-01,Collective

TCGA-AA-3811-01,Collective

TCGA-AA-3814-01,Collective

TCGA-AA-3815-01,Collective

TCGA-AA-3821-01,Collective

TCGA-AA-3831-01,Collective

TCGA-AA-3841-01,Collective

TCGA-AA-3845-01,Collective

TCGA-AA-3846-01,Collective

TCGA-AA-3850-01,Collective

TCGA-AA-3851-01,Collective

TCGA-AA-3852-01,Collective

TCGA-AA-3855-01,Collective

TCGA-AA-3856-01,Collective

TCGA-AA-3860-01,Collective

TCGA-AA-3862-01,Collective

TCGA-AA-3864-01,Collective

TCGA-AA-3869-01,Collective

TCGA-AA-3875-01,Collective

TCGA-AA-3930-01,Collective

TCGA-AA-3939-01,Collective

TCGA-AA-3947-01,Collective

TCGA-AA-3950-01,Collective

TCGA-AA-3955-01,Collective

TCGA-AA-3956-01,Collective

TCGA-AA-3968-01,Collective

TCGA-AA-3970-01,Collective

TCGA-AA-3971-01,Collective

TCGA-AA-3972-01,Collective

TCGA-AA-3975-01,Collective

TCGA-AA-3976-01,Collective

TCGA-AA-3977-01,Collective

TCGA-AA-3980-01,Collective

TCGA-AA-3982-01,Collective

TCGA-AA-3984-01,Collective

TCGA-AA-3986-01,Collective

TCGA-AA-3989-01,Collective

TCGA-AA-3994-01,Collective

TCGA-AA-A004-01,Collective

TCGA-AA-A00A-01,Collective

TCGA-AA-A00E-01,Collective

TCGA-AA-A00F-01,Collective

TCGA-AA-A00J-01,Collective

TCGA-AA-A00K-01,Collective

TCGA-AA-A00R-01,Collective

TCGA-AA-A017-01,Collective

TCGA-AA-A02F-01,Collective

TCGA-AA-A02H-01,Collective

TCGA-AA-A03J-01,Collective

TCGA-AD-6890-01,Collective

TCGA-AD-A5EJ-01,Collective

TCGA-AM-5821-01,Collective

TCGA-AU-3779-01,Collective

TCGA-AU-6004-01,Collective

TCGA-AY-4070-01,Collective

TCGA-AY-4071-01,Collective

TCGA-AZ-4315-01,Collective

TCGA-AZ-4615-01,Collective

TCGA-AZ-4616-01,Collective

TCGA-AZ-4682-01,Collective

TCGA-AZ-4684-01,Collective

TCGA-CK-4948-01,Collective

TCGA-CK-4950-01,Collective

TCGA-CK-4951-01,Collective

TCGA-CK-5912-01,Collective

TCGA-CK-5913-01,Collective

TCGA-CK-5914-01,Collective

TCGA-CK-6747-01,Collective

TCGA-CK-6751-01,Collective

TCGA-CM-4743-01,Collective

TCGA-CM-4748-01,Collective

TCGA-CM-4750-01,Collective

TCGA-CM-4751-01,Collective

TCGA-CM-4752-01,Collective

TCGA-CM-6171-01,Collective

TCGA-CM-6172-01,Collective

TCGA-CM-6678-01,Collective

TCGA-D5-6529-01,Collective

TCGA-D5-6533-01,Collective

TCGA-D5-6535-01,Collective

TCGA-D5-6536-01,Collective

TCGA-D5-6540-01,Collective

TCGA-D5-6920-01,Collective

TCGA-D5-6931-01,Collective

TCGA-D5-6932-01,Collective

TCGA-D5-7000-01,Collective

TCGA-DM-A0XD-01,Collective

TCGA-DM-A1HB-01,Collective

TCGA-DM-A280-01,Collective

TCGA-F4-6806-01,Collective

TCGA-F4-6808-01,Collective

TCGA-F4-6856-01,Collective

TCGA-G4-6293-01,Collective

TCGA-G4-6298-01,Collective

TCGA-G4-6322-01,Collective

TCGA-G4-6588-01,Collective

TCGA-NH-A50V-01,Collective

TCGA-QG-A5YW-01,Collective

TCGA-A6-2674-01,CryptLike

TCGA-A6-2675-01,CryptLike

TCGA-A6-2681-01,CryptLike

TCGA-A6-2682-01,CryptLike

TCGA-A6-2684-01,CryptLike

TCGA-A6-3808-01,CryptLike

TCGA-A6-3810-01,CryptLike

TCGA-A6-5664-01,CryptLike

TCGA-A6-6138-01,CryptLike

TCGA-A6-6142-01,CryptLike

TCGA-A6-6654-01,CryptLike

TCGA-A6-6781-01,CryptLike

TCGA-A6-A566-01,CryptLike

TCGA-AA-3489-01,CryptLike

TCGA-AA-3496-01,CryptLike

TCGA-AA-3532-01,CryptLike

TCGA-AA-3554-01,CryptLike

TCGA-AA-3684-01,CryptLike

TCGA-AA-3715-01,CryptLike

TCGA-AA-3833-01,CryptLike

TCGA-AA-3842-01,CryptLike

TCGA-AA-3858-01,CryptLike

TCGA-AA-3866-01,CryptLike

TCGA-AA-3872-01,CryptLike

TCGA-AA-3877-01,CryptLike

TCGA-AA-3949-01,CryptLike

TCGA-AA-3952-01,CryptLike

TCGA-AA-3966-01,CryptLike

TCGA-AA-A00N-01,CryptLike

TCGA-AA-A00O-01,CryptLike

TCGA-AA-A01D-01,CryptLike

TCGA-AA-A01P-01,CryptLike

TCGA-AD-6548-01,CryptLike

TCGA-AD-6895-01,CryptLike

TCGA-AD-6899-01,CryptLike

TCGA-AZ-4308-01,CryptLike

TCGA-AZ-5403-01,CryptLike

TCGA-AZ-6600-01,CryptLike

TCGA-AZ-6603-01,CryptLike

TCGA-AZ-6605-01,CryptLike

TCGA-AZ-6607-01,CryptLike

TCGA-CK-5916-01,CryptLike

TCGA-CK-6748-01,CryptLike

TCGA-CM-5341-01,CryptLike

TCGA-CM-5348-01,CryptLike

TCGA-CM-5349-01,CryptLike

TCGA-CM-5860-01,CryptLike

TCGA-CM-5862-01,CryptLike

TCGA-CM-5863-01,CryptLike

TCGA-CM-6162-01,CryptLike

TCGA-CM-6163-01,CryptLike

TCGA-CM-6168-01,CryptLike

TCGA-CM-6170-01,CryptLike

TCGA-CM-6674-01,CryptLike

TCGA-CM-6677-01,CryptLike

TCGA-CM-6679-01,CryptLike

TCGA-CM-6680-01,CryptLike

TCGA-D5-5538-01,CryptLike

TCGA-D5-5539-01,CryptLike

TCGA-D5-6923-01,CryptLike

TCGA-D5-6924-01,CryptLike

TCGA-D5-6926-01,CryptLike

TCGA-D5-6927-01,CryptLike

TCGA-D5-6928-01,CryptLike

TCGA-D5-6930-01,CryptLike

TCGA-F4-6463-01,CryptLike

TCGA-F4-6703-01,CryptLike

TCGA-F4-6805-01,CryptLike

TCGA-F4-6807-01,CryptLike

TCGA-F4-6854-01,CryptLike

TCGA-G4-6297-01,CryptLike

TCGA-G4-6303-01,CryptLike

TCGA-G4-6311-01,CryptLike

TCGA-G4-6627-01,CryptLike

TCGA-G4-6628-01,CryptLike

TCGA-WS-AB45-01,CryptLike

TCGA-3L-AA1B-01,EMT

TCGA-5M-AAT6-01,EMT

TCGA-A6-2671-01,EMT

TCGA-A6-2685-01,EMT

TCGA-A6-5656-01,EMT

TCGA-A6-5657-01,EMT

TCGA-A6-5659-01,EMT

TCGA-A6-5667-01,EMT

TCGA-A6-6141-01,EMT

TCGA-A6-6651-01,EMT

TCGA-A6-6782-01,EMT

TCGA-A6-A5ZU-01,EMT

TCGA-AA-3514-01,EMT

TCGA-AA-3527-01,EMT

TCGA-AA-3812-01,EMT

TCGA-AA-3867-01,EMT

TCGA-AA-3870-01,EMT

TCGA-AA-A00D-01,EMT

TCGA-AA-A01K-01,EMT

TCGA-AA-A01X-01,EMT

TCGA-AA-A02R-01,EMT

TCGA-AD-5900-01,EMT

TCGA-AD-6901-01,EMT

TCGA-AD-6964-01,EMT

TCGA-AM-5820-01,EMT

TCGA-AY-6196-01,EMT

TCGA-AZ-4323-01,EMT

TCGA-AZ-6601-01,EMT

TCGA-CA-5254-01,EMT

TCGA-CA-5797-01,EMT

TCGA-CA-6717-01,EMT

TCGA-CA-6718-01,EMT

TCGA-CA-6719-01,EMT

TCGA-CM-4747-01,EMT

TCGA-CM-5344-01,EMT

TCGA-CM-5868-01,EMT

TCGA-CM-6165-01,EMT

TCGA-CM-6167-01,EMT

TCGA-CM-6169-01,EMT

TCGA-D5-5541-01,EMT

TCGA-D5-6531-01,EMT

TCGA-D5-6534-01,EMT

TCGA-D5-6541-01,EMT

TCGA-D5-6898-01,EMT

TCGA-D5-6922-01,EMT

TCGA-D5-6929-01,EMT

TCGA-F4-6459-01,EMT

TCGA-F4-6460-01,EMT

TCGA-F4-6461-01,EMT

TCGA-F4-6569-01,EMT

TCGA-F4-6570-01,EMT

TCGA-F4-6704-01,EMT

TCGA-F4-6809-01,EMT

TCGA-F4-6855-01,EMT

TCGA-G4-6299-01,EMT

TCGA-G4-6302-01,EMT

TCGA-G4-6310-01,EMT

TCGA-G4-6314-01,EMT

TCGA-G4-6625-01,EMT

TCGA-NH-A6GC-01,EMT

TCGA-NH-A8F8-01,EMT

TCGA-QG-A5Z1-01,EMT
