# Supplementary figures and images for: Based on Molecular Subtypes, Immune Characteristics and Genomic Variation to Constructing and Verifying Multi-Gene Prognostic Characteristics of Colorectal Cancer
Source: Front Cell Dev Biol. 2022 Feb 23;10:828415. doi: 10.3389/fcell.2022.828415 (PMC8905350; doi:10.3389/fcell.2022.828415)

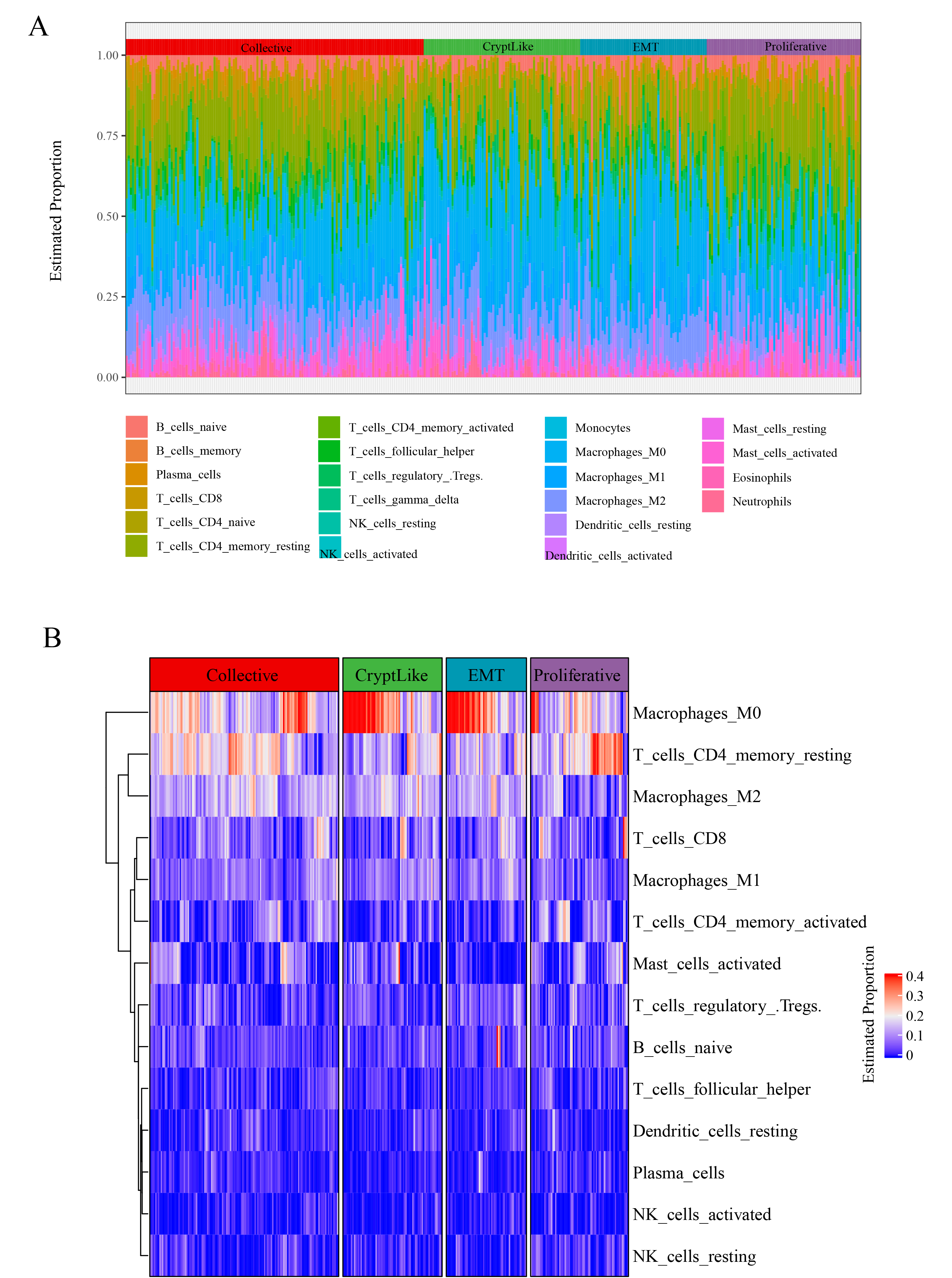

Supplement: Supplementary file 2 [file Image1.JPEG]
